# Supplementary material for: Progress in Nonalcoholic Fatty Liver Disease: SIRT Family Regulates Mitochondrial Biogenesis
Source: Biomolecules. 2022 Aug 5;12(8):1079. doi: 10.3390/biom12081079 (PMC9405760; doi:10.3390/biom12081079)
Supplement: Supplementary file 1 [file biomolecules-12-01079-s001.zip › biomolecules-1833852-supplementary.pdf]

Supplementary Material

Table S1. The function and mechanism of SIRT in regulation of mitochondrial biogenesis with NAFLD.

Reference	Country	Publication Year	Stimulation	Active State of SIRT	Molecules Involved	Effects
Yang M [110]	China	2021	conditioned medium	upregulated protein levels of SIRT1	PGC-1α	Improved insulin resistance in diabetic mice, amended the pathological structure of the liver, enhanced the liver's total antioxidant capacity and mitochondrial function, reduced inflammation and cell apoptosis.
Jiang Y [114]	China	2021	HFD	decreased the mRNA and protein levels of SIRT1	PGC-1α, Beclin-1, p62/SQSTM1	Caused viability reduction, apoptosis, lipid accumulation, and reactive oxygen species production.
Yang H [111]	China	2021	SIRT1-knockdown db/m mice	decreased the mRNA and protein levels of SIRT1	PGC-1α	Increased oxidative stress and mitochondrial dysfunction.
Yang YL [115]	China	2020	MicroRNA-29a, MCD diet	decreased the mRNA and protein levels of SIRT1	GSK3β, PGC-1α, TFAM, CHOP, LONP1, CD36	Reversed the aberrant SIRT1-mediated mitochondrial biogenesis and mitochondrial proteostatic stress, ultimately mitigating the pathological progression.
Ren T [122]	China	2019	blueberry juice	upregulated protein levels of SIRT1	PGC-1α, GSH, SOD2	Reduced mitochondrial oxidative stress, decreasing the MDA level, elevated the GSH and SOD levels, and suppressed the ROS activity.
da Silva Lima N [21]	Spain.	2022	knockdown of ATG3	upregulated protein levels of SIRT1	JNK1, CPT1a	Ameliorated p63- and diet-induced steatosis, improved fatty acid metabolism and stimulated mitochondrial function.
He X [117]	China	2019	Cadmium	suppressed SIRT1 signaling pathway	PPARα, CPT1a, VLCAD, MCAD	Inhibited the mitochondrial fatty acid oxidation.

Guo L [45]	China	2019	high-fat/high-sucrose diet-fed mice	decreased the mRNA and protein levels of SIRT2	ACLY	Overexpressing SIRT2 in AML12 cells inhibited lipid accumulation, which was reversed by overexpressing the ACLY-3KQ variant rather than by overexpressing WT ACLY.
Du S [140]	China	2022	curcumin	upregulated the mRNA and protein levels of SIRT3	SOD2	Decreased the levels of cellular lipids and mitochondrial reactive oxygen species, and increased the mitochondrial DNA copy number and superoxide dismutase activity in fatty L02 cells.
Zhang X [146]	China	2021	Major royal jelly proteins	upregulated the mRNA and protein levels of SIRT3	AMPK, SOD2, COX IV	Reduced lipid droplet content, triglyceride, ALT, and AST, and restored mitochondrial membrane potential.
Zeng X [145]	China	2019	dihydromyricetin	upregulated the mRNA and protein levels of SIRT3	AMPK, PGC-1α, ERRα	Ameliorated mitochondrial dysfunction and oxidative stress.
Cheng Y [150]	China	2016	microRNA-421	decreased the mRNA and protein levels of SIRT3	FOXO3, MnSOD, CAT	Modulated the oxidant stress and lipid metabolism, and reduced celluar oxidative damage.
Teodoro JS [151]	Portugal	2013	Berberine	Increased mitochondrial SIRT3 activity	COX, SDH	Increased mitochondrial SIRT3 activity, normalized mitochondrial function, and prevented a state of energetic deficit.
He J [141]	USA	2013	Activation of AhR, MCD diet	deactivated the mitochondrial SIRT3	SOD2, TiPARP	Increased hepatic steatosis, production of ROS, and lipid peroxidation.
Valdecantos MP [152]	Spain	2012	Lipoic acid	Increased mitochondrial SIRT3 activity	Foxo3a, PGC-1β	Prevented hepatic triglyceride accumulation and liver oxidative damage and stimulation of mitochondrial antioxidant defenses.

Sun M [147]	USA	2021	cocoa or cocoa polyphenols	upregulated the protein levels of SIRT3	PGC1a, NRF1, Foxo3a	Prevented body weight gain, hepatic triacylglycerols, lipid peroxides, and mitochondrial DNA damage.
Staňková P [154]	Czech Republic	2021	Western-style diet	decreased the mRNA and protein levels of SIRT3	SDH, SUCNR1	Did not observe signs of oxidative damage compared to the control group and may be adaptive to prevent mitochondrial respiratory chain overload and massive ROS production.
Nassir F [153]	USA	2018	Overexpression of SIRT3 in MTP+/- mice, HFD diet	upregulated the mRNA and protein levels of SIRT3	MTP, FAO, CD38	Reduced the acetylation of MTP compared with β-galactosidase controls, increased mitochondrial FAO, and reduced hepatic steatosis, CD68, and serum ALT levels.
Liu Y [155]	China	2016	RBP4-Tg mice	decreased activity of SIRT3	long-chain acyl-coenzyme A dehydrogenase	Induced mitochondrial dysfunction and the deterioration of lipid metabolism.
Guo L [158]	China	2016	knockdown of SIRT4 in the livers of HF/HS diet-fed mice	decreased the mRNA and protein levels of SIRT4	MTPα,	Promoted hepatic fatty acid oxidation, as indicated by elevated plasma β-hydroxybutyrate and inhibited hepatic steatosis and its accompanying pathology and metabolic disorders.
Zhang S [168]	China	2022	2,3,5,4′-tetrahydroxy-stilbene-2-O-β-d-glucoside	increased SIRT5 expression by regulating its mRNA stability through enhancing the binding of SIRT5 mRNA with serine/arginine-rich splicing factor 2	CPT1A,SRSF2	Reduced ROS formation and attenuated mitochondrial dysfunction both in vivo and in vitro.
Goetzman ES [167]	USA	2020	Sirt5 knockout mice, Medium-chain triglycerides	decreased the mRNA and protein levels of SIRT5	mitochondrial acyl-CoA synthetase, mitochondrial C12	Induced the steatosis phenotype.

Abbreviations: PGC-1α, peroxisome proliferator-activated receptor gamma coactivator-1α; p62: p62/SQSTM1, sequestosome 1; GSK3β, glycogen synthase kinase 3 beta; TFAM, mitochondrial transcription factor A; CHOP, C/EBP homologous protein; LONP1, lon peptidase 1; JNK1, Jun N-terminal protein kinase 1; CPT1a, carnitine palmitoyltransferase 1A; PPARα, peroxisome proliferator–activated receptor α; ACLY, adenosine triphosphate-citrate lyase; AMPK, adenosine 5-monophosphate (AMP)-activated protein kinase; COX IV, cytochrome c oxidase IV; ERRα, FOXO3, Forkhead Box O3; CAT, catalase; SOD, superoxide dismutase; TiPARP, tetrachlorodibenzo-p-dioxin (TCDD)-inducible poly(ADP-ribose) polymerase; NRF1, nuclear respiratory factor 1; SDH, succinate dehydrogenase; SUCNR1, Succinate receptor 1; MTP, mitochondrial trifunctional protein; FAO, fatty acid oxidation; SRSF2, serine/arginine-rich splicing factor 2.
